# Gas Alert: The NO_2_ Pitfall during NO Fumigation of Plants

**DOI:** 10.3389/fpls.2017.00085

**Published:** 2017-01-31

**Authors:** Dörte Kasten, Jörg Durner, Frank Gaupels

**Affiliations:** Institute of Biochemical Plant Pathology, Helmholtz Zentrum München, German Research Center for Environmental HealthNeuherberg, Germany

**Keywords:** nitric oxide, nitrogen dioxide, gas, fumigation, response

## Introduction

Physiological functions of nitric oxide (NO) in plants are often investigated by using chemical NO donors (Feelisch, 1998; Floryszak-Wieczorek et al., 2006) or fumigation of plants with NO gas (Huang et al., 2004; Palmieri et al., 2008; Vitor et al., 2013; Frungillo et al., 2014; Kasten et al., 2016; Krasuska et al., 2016; León et al., 2016; Melo et al., 2016). Treatment with gaseous NO has the advantage of being non-invasive and time- as well as cost effective. However, NO can react with air-oxygen resulting in the rise of toxic nitrogen dioxide (NO_2_; Groß et al., 2013; Heinrich et al., 2013). In human medicine NO_2_ formation is a well-established risk factor during NO inhalation as a cure against pulmonary diseases (Schedin et al., 1999; Sokol et al., 1999). The damaging effect of NO_2_ on plants has been frequently demonstrated (Wellburn, 1990; Xu et al., 2010; Liu et al., 2015; Kasten et al., 2016). In this current opinion article fumigation approaches were critically re-evaluated with a special focus on contaminations of NO-enriched air with NO_2_. Potential artifacts and data misinterpretation due to unintended co-treatment of plants with both gases are highlighted.

## NO fumigation

The chemistry of NO donors is rather complex. For instance, the commonly used NO donors sodium nitroprusside, S-nitroso-N-acetyl-penicillin, and S-nitrosoglutathione do not only release various NO derivatives but also cyanide ions, N-acetyl-penicillin, and oxidized glutathione, respectively (Feelisch, 1998). All of these compounds could evoke specific responses in plant cells and therefore have to be evaluated carefully by appropriate control treatments.

By contrast, NO fumigation loads the leaf mainly with NO and nitrite that also emerge under natural conditions e.g., during stress signaling (Ignarro et al., 1993; Groß et al., 2013). In the aqueous environment of a cell both nitrogen oxides are in equilibrium, with nitrous acid as an unstable intermediate. Nitrite is either converted to NO or is efficiently scavenged by the enzyme nitrite reductase. Accordingly, fumigation with 30 parts per million (ppm) NO for 1 h did not induce nitrite accumulation in leaves of Arabidopsis (*Arabidopsis thaliana*; Kasten et al., 2016). Gaseous NO enters leaves via the stomata although NO as a lipophilic molecule can also penetrate the cuticle to a certain extent (Wellburn, 1990). This facilitates the non-invasive treatment of many plants in parallel such as in the course of a mutant screen (Kasten et al., 2016). Another advantage of gaseous NO is the possibility of its continuous application over long time periods e.g., during pathogen infection (Vitor et al., 2013). In any case, it is necessary to determine the plants stomatal conductance under the prevailing experimental conditions. Hereby, an equal uptake of NO by the plants is ensured, especially when different mutant lines are compared (Kasten et al., 2016).

Fumigations of plants with high concentrations of NO are usually done either in closed- or flow-through chambers. A very basic set-up for a closed system would consist of a plant placed in a sealed container filled with (NO-free) air. Dependent on the volume of the headspace, an appropriate dose of NO (usually formulated in N_2_) would be injected into the chamber to adjust the desired NO concentration (Huang et al., 2004; Palmieri et al., 2008; León et al., 2016). Alternatively, a NO-releasing nitrite/hydrogen chloride solution could be placed in the container alongside the plants (Krasuska et al., 2016).

**Figure 1:**
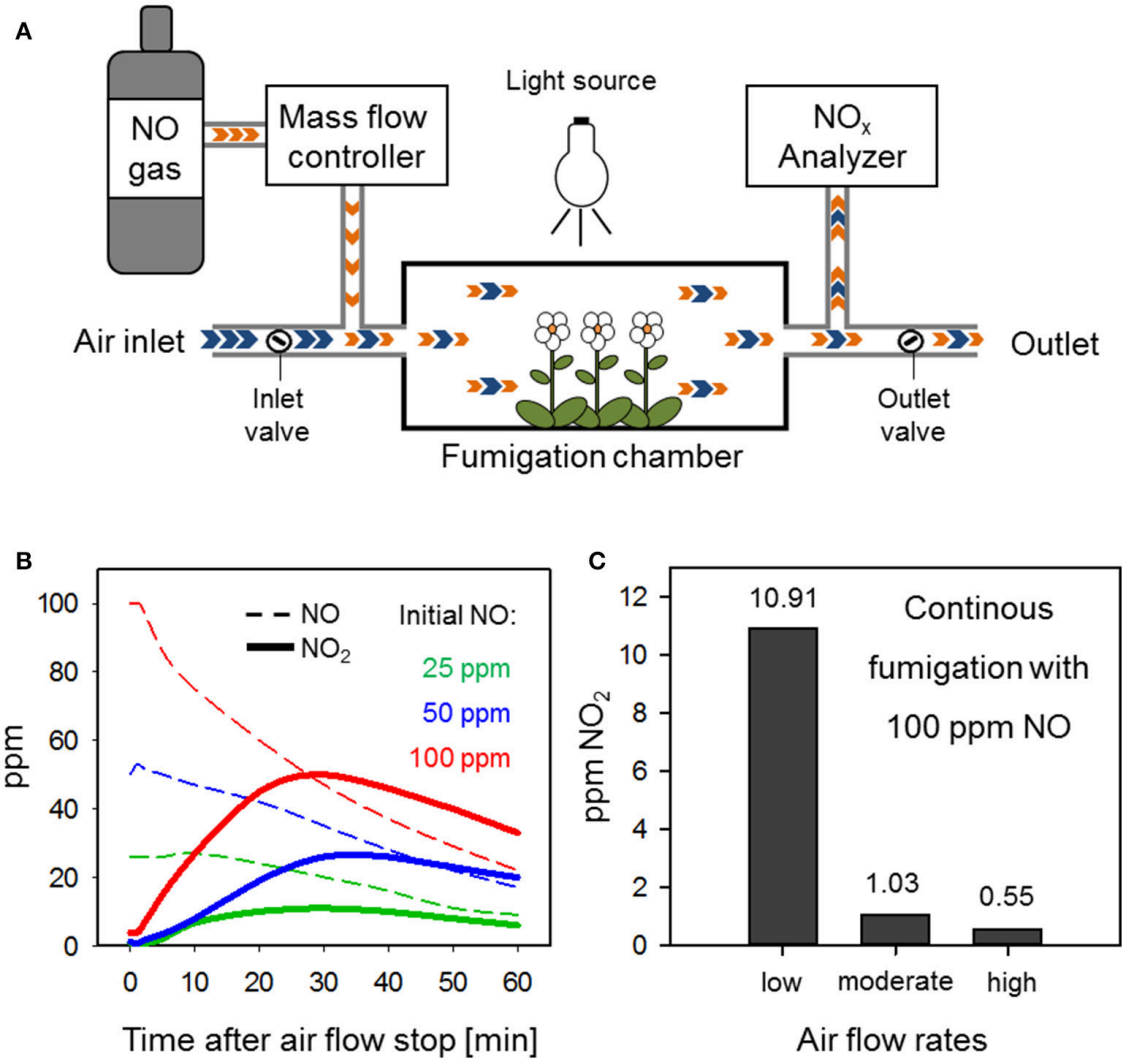
**An adequate set-up of a flow-through system for NO fumigation prevents NO_2_ formation. (A)** Schematic diagram of an advanced flow-through system for NO fumigation. Blue arrows, air; orange arrows, NO. **(B)** NO_2_ accumulation in a fumigation chamber with disrupted air flow. Different initial NO concentrations (25 ppm = green, 50 ppm = blue, 100 ppm = red) where adjusted within the chamber before stopping the air flow (*t* = 0 min) by shutting the inlet and outlet valves. Changes in NO (dotted lines) and NO_2_ (solid lines) were measured over 60 min via an NO_x_ Analyzer. **(C)** NO_2_ formation in a flow-through system is dependent on air flow rates. The NO concentration within the chamber was set to 100 ppm NO under low, moderate, or high air flow rate conditions and NO_2_ accumulation was measured 30 min after the flow-through system was equilibrated.

An advanced flow-through system for fumigation with NO is shown in Figure 1A (Kasten et al., 2016). Plants are placed into an air-tight fumigation chamber which is set to appropriate illumination and temperature conditions. A constant and manipulable air flow is realized by adjustable inlet and outlet air flows. Here, the air withdrawal (outlet air flow) from the chamber should exceed the air intake (inlet air flow) to cause slight negative pressure. By regulating the inlet and outlet air flow, the flow rate within the chamber can be manipulated. The air is charged with NO (here 15% NO in N_2_) upstream of the fumigation chamber at a distance that ensures proper mixing of the gases. NO volumes introduced to the system per time unit (e.g., ml/min) are regulated by a mass flow controller. The final NO concentration within the chamber is monitored by branching off the outlet flow to an AC32M NO analyzer (Environment S.A.) when it exits the chamber. This device is able to determine the amount (ppm) of NO, NO_2_, and the sum of both (NO_x_) within an air sample.

## NO_2_ formation during NO fumigation

A risk of fumigations with high levels of NO is the concomitant emergence of NO_2_. Autoxidation of NO in the presence of molecular oxygen (according to the formula 2NO + O_2_ → 2NO_2_) exhibits a second-order dependence on NO concentration and, therefore, is a slow reaction at low but a rapid reaction at high NO levels (Schedin et al., 1999; Sokol et al., 1999; Heinrich et al., 2013). Disruption of the air flow through the NO fumigation chamber shown in Figure 1A by closing the inlet and outlet valves caused the accumulation of NO_2_ within a few minutes (Figure 1B). At a starting concentration of 100 ppm NO the level of NO_2_ reached 15 ppm at 5 min and 27 ppm at 10 min after stopping the flow. Initial 25 ppm NO resulted in the formation of 2 and 7 ppm NO_2_ at 5 and 10 min after chamber closure (Figure 1B). In comparison, other researchers exposed Arabidopsis to 300 ppm NO for 5 min (León et al., 2016) or even 1250 ppm NO for 10 min (Huang et al., 2004; Palmieri et al., 2008) in closed containers. Under such conditions a significant build-up of NO_2_ can be expected.

In flow-through systems the air/O_2_/NO mixture is continuously exchanged which limits the reaction time for autoxidation of NO. Hence, it depends on the flow rate how much NO_2_ is formed in the system. The relationship between flow rate and NO_2_ formation is illustrated in Figure 1C. Here, a constant level of 100 ppm NO was accompanied by 1.03 ppm NO_2_ at moderate, 10.9 ppm NO_2_ at low but only 0.55 ppm NO_2_ at high air flux rates. The aforementioned NO_2_ levels were already formed 5 min after the start of fumigation and remained stable over the next 30 min (data not shown). Baseline NO_2_ levels of 0.05 ppm were measured in air that was not charged with NO (data not shown). These results suggest that previously applied NO concentrations between 50 and 150 ppm (Frungillo et al., 2014; Melo et al., 2016) could readily react with air-O_2_ leading to the formation of contaminating NO_2_ in the upper parts per billion (ppb) to low ppm range dependent on air flow. As compared to closed chamber systems, the rate of NO_2_ formation is rather low in flow-through systems. However, long-term exposure to ppb levels of NO_2_ can still have a profound impact on plants as discussed in the next chapter.

It is worth mentioning that long-term storage of commercial NO gas in pressurized cylinders can cause a substantial accumulation of NO_2_ due to NO conversion under high pressure to NO_2_ and N_2_O (Tsukahara et al., 2002). Such findings emphasize again the need for careful monitoring of NO_2_ during NO fumigations.

## NO_2_ and NO induce distinct but overlapping responses

Recently, a highly controlled fumigation system was employed for comparing responses of Arabidopsis to ppm levels of NO and NO_2_ (Kasten et al., 2016). The nitrite content was strongly increased after NO_2_ but decreased after NO exposure. Fumigation for 1 h with 20 or 30 ppm NO_2_ triggered rapid lesion formation that was dependent on NO and hydrogen peroxide (Kasten et al., 2016). By contrast, neither 30 ppm NO for 1 h (Kasten et al., 2016) nor 60 ppm NO for 12 h (Vitor et al., 2013; Frungillo et al., 2014) or even 50–150 ppm NO for up to 72 h (Melo et al., 2016) led to any visible leaf damage in Arabidopsis and tomato (*Solanum lycopersicum*). Collectively, these results demonstrate that NO_2_ and NO have distinct chemistry and toxicity within the leaf.

Exposure of Arabidopsis plants to 300 ppm NO for 10 min in a closed chamber resulted in cell death, protein tyrosine nitration, oxylipin accumulation, and ascorbate depletion (León et al., 2016). Importantly, all of these effects were also observed after treatment with 30 ppm NO_2_ but not 30 ppm NO in a flow-through fumigation system (Kasten et al., 2016). Moreover, gaseous NO administered in a sealed vessel regulated a disparate set of genes than the NO donor NOR3 (NO-releasing agent-3; Palmieri et al., 2008). This further supports the assumption that at least some of the observed plant responses to NO fumigation in closed chamber systems were actually induced by the unnoticed rise of NO_2_ under such conditions.

Long-term fumigation of plants with NO_2_ can induce growth and leaf greening at ppb levels (Srivastava et al., 1994; Takahashi et al., 2014) or antioxidant defense, severe stress responses, and leaf damage at low ppm levels (Xu et al., 2010; Liu et al., 2015) dependent on the sensitivity of the plant species investigated. In this regard, it would be of interest if some of these plant responses that were also reported after long-term fumigations with NO were actually mediated by the concomitant formation of NO_2_. For instance, fumigation for 24–72 h with 50–150 ppm NO triggered the biosynthesis of chlorophyll and carotenoids in greening tomato seedlings (Melo et al., 2016). Previous work revealed an elevated chlorophyll and total nitrogen content in bean (*Phaseolus vulgaris*) grown for 5 days in an atmosphere containing 0.3 ppm NO_2_ (Srivastava et al., 1994). Other researchers found that carotenoid antioxidants efficiently scavenge NO_2_ *in vitro* and *in vivo* in human leucocytes (Cooney et al., 1994; Böhm et al., 1995). Thus, it seems feasible that chlorophyll and carotenoid biosynthesis are activated by NO_2_. The involvement of NO in this process remains to be elusive due to a lack of convincing evidence.

## Conclusions

High (ppm) levels of NO efficiently react with air-O_2_ to give NO_2_. This must be considered when planning treatments of plants with gaseous NO. Particularly in closed chamber systems without air flow, NO_2_ strongly accumulates within a few minutes. Even in advanced flow-through systems high NO levels are often accompanied by NO_2_ concentrations known to trigger stress responses in plants. Actually, in many published studies it is inconclusive if NO_2_ rather than NO was the bioactive compound within the applied gas mixture. Therefore, the central message of the current opinion paper is a strong recommendation to monitor NO_2_ during NO fumigation. This would improve the interpretation and reproduction of published results from NO fumigation experiments. If NO and NO_2_ cannot be determined, the respective treatments should be referred to as “NO_x_ fumigation,” and NO_2_ should be discussed as a potential inducer of the observed plant responses.

## Author contributions

DK and FG did the fumigation experiments. DK, JD, and FG wrote the manuscript.

### Conflict of interest statement

The authors declare that the research was conducted in the absence of any commercial or financial relationships that could be construed as a potential conflict of interest.
